# Milk yield, rumen fermentation, and microbiota of Shami goats fed diets supplemented with spirulina and yeast

**DOI:** 10.1186/s13568-025-01916-3

**Published:** 2025-07-21

**Authors:** Alaa Emara Rabee, Moustafa Mohamed M. Ghandour, Ahmed M. Sallam, Osama Raef, Eman A. Elwakeel, Ebrahim A. Sabra, Adel M. Abdel-Wahed, Salah Abo Bakr, Hanan Saad ElSamahy, Amal Amin Hamed, Mebarek Lamara

**Affiliations:** 1 Animal and Poultry Nutrition Department, Desert Research Center, Ministry of Agriculture and Land Reclamation, Cairo, Egypt; 2 Animal and Poultry Breeding Department, Desert Research Center, Ministry of Agriculture and Land Reclamation, Cairo, Egypt; 3 Department of Animal and Fish Production, Faculty of Agriculture, Alexandria University, Alexandria, Egypt; 4 Genetic Engineering and Biotechnology Research Institute, University of Sadat City, Sadat City, Egypt; 5 Animal and Poultry Health Department, Desert Research Center, Ministry of Agriculture and Land Reclamation, Cairo, Egypt; 6 Botany and Microbiology Department, Faculty of Science, Cairo University, Cairo, Egypt; 7 Forest Research Institute, University of Quebec in Abitibi-Temiscamingue, Rouyn-Noranda, Canada

**Keywords:** Shami goats, Milk yield and feed efficiency, *Spirulina* and yeast, Rumen, Fermentation, Bacteria and archaea

## Abstract

Microalgae and live yeast have gained interest in improving animal performance. This study evaluated the effect of supplementation with *Spirulina platensis*, *Saccharomyces cerevisiae*, or their combination on the in vitro and in vivo rumen fermentation, rumen microbiota, and milk yield and composition of lactating Shami goats. The in vitro experiment included four diets: non-supplemented basal diet consisted of Alfalfa hay and a concentrate feed mixture (C); basal diet supplemented with 1% *Saccharomyces* (Y) based on dry matter; basal diet supplemented with 1% *Spirulina* (A); and basal diet supplemented with 1% of a mixture of *Saccharomyces* and *Spirulina* (AY). In the in vivo experiment, twenty-one lactating goats were divided into three groups (n = 7) to receive one of three diets: C, A, and AY. Group AY had higher in vitro gas production, dry matter digestion (DMD), and volatile fatty acids (VFA) (*p* < 0.05). Milk yield and feed efficiency were higher in groups A and AY compared to group C. Group AY goats exhibited higher rumen total VFA, acetic, and propionic, while group A showed higher butyric acid. Lower predicted methane was observed in group AY. Groups A and AY showed distinctive microbial communities. The bacterial community was dominated by phylum Bacteroidota, and genera *Prevotella* and Rikenellaceae RC9 gut group, which were higher in the AY group. The archaeal community was dominated by the genus *Methanobrevibacter*, which had a lower prevalence in group AY. The combination of live yeast and *Spirulina* improved rumen fermentation and the milk yield; therefore, it could be used as a feed additive for lactating goats.

**Supplementary Information:**

The online version contains supplementary material available at 10.1186/s13568-025-01916-3.

## Introduction

Goats contribute substantially to food security in the world, as the increase in world population and climate change occur. Shami (also known as Damascus) goat breed is one of the main goat breeds in Mediterranean countries due to their high potential for fertility and milk production under harsh conditions (Alsheikh 2013; Almasri et al. 2023). The main challenges that face the intensification of animal production in arid countries include low feed availability and health problems (Xue et al. 2022). Therefore, high-quality feed additives are required to improve rumen fermentation and animal productivity.

Rumen fermentation relies on interaction between complex networks of microbial groups, including bacteria, archaea, protozoa, and fungi. These microorganisms convert plant materials into VFAs and microbial proteins that provide the host animal with the majority of its energy and protein requirements (Rabee et al. 2022). Rumen fermentation also produces different gases that are dominated by methane, which represents a loss in the animal’s gross energy feed intake and causes climate change (Brooke et al. 2019). Consequently, improving the efficiency of microbial fermentation in the rumen will improve animal’s efficiency and reduce methane emissions from the livestock sector.

Probiotics such as *Saccharomyces cerevisiae* and prebiotics such as the products of microalgae, including *Spirulina platensis,* represent promising feed supplements that are rich in nutrients (> 35% crude protein) and bioactive compounds that stimulate rumen fermentation to increase animal productivity (Grimm et al. 2020). *Spirulina platensis* can impact the rumen microbial fermentation and animal performance due to the presence of nutrients such as protein, essential amino acids, unsaturated fatty acids, vitamins, and minerals, as well as bioactive compounds such as phenols, flavonoids, and saponins. These phytochemicals modulate rumen microbiota and improve rumen fermentation (Finamore et al. 2017). Christodoulou et al. (2023) reported that *Spirulina* supplementation in ewes enriched rumen cellulolytic bacteria such as *Fibrobacter succinogenes*, and *Ruminococcus albus* but decreased the amylolytic bacteria such as *Ruminobacter amylophilus,* and *Selenomonas ruminantium*. Similarly, Spirulina supplementation in lambs, improved the concentration of VFA and rumen papilla length as well as bacterial genus *Prevotella* and *Megasphaera* (Wang et al. 2023). Supplementation the lactating ewes and cattle with *Spirulina platensis* improved milk yield (Kulpys et al. 2009; El-Deeb et al. 2023).

*Saccharomyces* optimizes anaerobic rumen fermentation through scavenging oxygen and stabilizes rumen pH by competing with lactic-producing bacteria for the available sugars, and promoting the lactic-utilizing bacteria (Zhang et al. 2023). Previous studies reported that *Saccharomyces* supplementation in different ruminant species improved milk yield and milk fat and lactose, and ruminal fiber-degrading bacteria such as *Christensenellaceae_R-7_group* and *Ruminococcaceae* (Mašek et al. 2008; Hadhoud et al. 2022; Zhang et al. 2023).

Combining microalgae with probiotics could support the growth of probiotics to improve the performance of host animals by stimulating the gut microorganisms (Gupta et al. 2017; Markowiak and Śliżewska 2018). Rabee et al. (2022) reported that camels and sheep supplemented with a combination of *Saccharomyces*, *Spirulina platensis*, and *Chlorella vulgaris* had enriched fiber-degrading bacteria and improved rumen fermentation, feed intake, and fiber digestibility of fibrous forage. Furthermore, Grimm et al. (2020) noticed that live yeast and microalgae (*Aurantiochytrium limacinum*) supplementation improved the fibrolytic functions in the gut of horses.

Limited information is available on the effect of a combination of live yeast and *Spirulina platensis* on rumen bacteria and archaea and milk production in goats. It was hypothesized that the combination of *Spirulina platensis* and *Saccharomyces cerevisiae* could be more effective in improving the efficiency of farm animals than *Spirulina* alone or live yeast alone (Rabee et al. 2022; Markowiak and Śliżewska 2018). Therefore, the objectives of this study were to assess the impact of *Spirulina platensis* or *Saccharomyces cerevisiae,* or their combination on in vitro and in vivo rumen fermentation, rumen microbiota (bacteria and archaea), milk production and composition, and feed efficiency of Shami goats.

## Materials and methods

### Ethics

The experiment was conducted under the guidelines of the Animal Care and Use Committee in the Division of Animal and Poultry Production, Desert Research Center, Egypt, and Research Ethics Review Committee, Faculty of Agriculture, University of Alexandria, Egypt (Reference: Alex. Agri. 082305307). All methods and protocols in this study comply with the ARRIVE 2.0 guidelines and the EU standards for the protection of animals. The sample size was decided based on the availability of animals with similar physical and physiological status. The study does not include clinical trials, and the experiment does not include animal euthanasia; and all animals were released to the goat herd after the end of the experiment.

### In vitro evaluation of experimental diets

This study was carried out at Maryout Research Station, Desert Research Center, Alexandria, Egypt. Four diets were used in this study as follows (Supplementary Table S1): Control diet consisted of 70% concentrate feed mixture (CFM) and 30% Alfalfa hay (*Medicago sativa*) (C); control diet supplemented with 1% of *Saccharomyces cerevisiae* on the dry matter (DM) basis (Y); control diet supplemented with 1% *Spirulina platensis* (A); and control diet supplemented with 1% of a mixture of *Saccharomyces* and *Spirulina* (50% *Saccharomyces* and 50% *Spirulina*) (AY) according to the recommendation of Rabee et al.(2022). The live yeast (*S. cerevisiae*, 2 * 10^7^ colony-forming units (CFU/g) is the product of the Angel yeast company (Angel yeast, Basatin, Cairo, Egypt). *Spirulina* is a commercial product of the Algal Biotechnology Unit, National Research Centre, Giza, Egypt. The proximate chemical compositions of CFM and Alfalfa hay are presented in Table 1. The chemical composition of *Spirulina platensis* is illustrated in Supplementary File S1.

**Table 1:** The chemical compositions of concentrate feed mixture and Alfalfa hay on the dry matter basis (g/kg)

	OM	CP	EE	NDF
Concentrate feed mixture (CFM)*	937.50	200.00	32.00	384.00
Alfalfa hay	907.70	146.40	20.30	498.80

*The concentrate mixture consisted of 56% corn, 23% soybean meal, 16% Wheat bran, 2.5% Cotton meal, 1.05% limestone, 0.75% salt, 0.1% sodium bicarbonate, 0.5% premix, and 0.1% antitoxins. OM = organic matter; CP = crude protein; EE = ether extract; NDF = neutral detergent fiber

The experimental rations were evaluated for gas production, fermentation parameters (pH, ammonia, VFA), and dry matter digestion (DMD). Rumen fluid was collected before morning feeding from three ruminally-cannulated rams fed alfalfa hay. Rumen fluids were filtered through four layers of cheesecloth. The incubation medium was prepared according to the method described by Menke et al. (1979). Four serum bottles were used for each diet, accompanied by four blank bottles (without substrates). The tested diets (200 mg) were added to the 125 mL serum bottles, and 40 mL of a mixture of 1 (rumen fluid):3 buffer solution (volume/volume) was tubed into the serum bottles. The bottles were sealed and incubated anaerobically at 39 °C for 24 h. Total gas production was recorded using graduated syringe displacement, and the values were corrected for the blank value. The gas yield was expressed as mL per 200 mg of DM. After 24 h of fermentation, all bottles were filtered in fiber filter bags with 25-micron porosity (ANKOM, Macedon, New York, United States), and the solid material was oven-dried at 60 °C and weighed to determine the DMD.

### In vivo experiment

#### Animals and diets

This study was carried out at Maryout Research Station, Desert Research Center, Alexandria, Egypt. The goats that were used in this study were the offspring of the experimental goat herd at Maryout Research Station, Desert Research Center, Alexandria, Egypt, and the animals were used in the experiment using the required consent from the administration of Maryout Research Station and the Animal and Poultry Production Division. Animals in this study were healthy animals, from the same goat breed (Shami), and had similar age, weight, milking stage, and milk yield. Twenty-one lactating Shami goats (34.85 ± 0.79 kg initial body weight and 3–4 years of age) in the early lactation stage (14 ± 7 days after kidding) were used in this 60-day experiment.

All the animals received the same basal diet that consisted of a 70% concentrate mixture and 30% Alfalfa hay (*Medicago sativa*) to meet the lactation feeding requirements for goats according to the National Research Council (NRC 2007). Goats were housed in shaded pens with free access to water and divided into three groups (n = 7) (Supplementary Table S1). The first goat group received the basal diet with no supplementation (C), the second goat group received a basal diet and supplemented with 1% of *Spirulina* on the DM basis (A), and the goat group received a basal diet and supplemented with 1% of a mixture of live yeast and Spirulina (50% live yeast and 50% Spirulina) (AY). The supplementation was mixed with the concentrate daily before feeding to confirm full intake. The body weight of goats was recorded every 15 days, and the lambs were reared with their dams, except for the days of milk yield estimation. The lambs had no access to dam diets but had free access to water. At the end of the experiment, all animals were released to the goat herd without euthanasia.

#### Rumen sampling and fermentation parameters

By the end of the experimental period, rumen fluid samples were collected from the animals using a stomach tube 3 h after feeding and strained through two layers of cheesecloth. The pH of rumen fluid was measured via a digital pH meter (WPA CD70, ADWA, Szeged, Hungary), and rumen samples were used to determine rumen VFA and ammonia, as well as microbial DNA extraction. To analyze ammonia and VFA, 1 mL of rumen sample was acidified with 200 μL of meta-phosphoric acid 25% (weight/volume). Then, the samples were centrifuged at 15,000 rpm for 20 min, and the supernatant was used for VFA and ammonia determination. Rumen ammonia (NH_3_-N) was measured calorimetrically using ammonia assay kits (Biodiagnostic, Cairo, Egypt). VFAs were measured by a gas chromatography system (TRACE 1300, Thermo Fisher Scientific, Waltham, United States) using a capillary column (TR-FFAP 30m × 0.53 mmI D × 0.5μm), and nitrogen was used as the carrier gas. The calibration was done using a standard with known concentrations of VFAs. The predicted methane yield was calculated according to Williams et al. (2019) using the concentration of propionic acid, Methane yield = 316/propionate + 4.4.

#### Milk yield and composition

On days 15th, 30th, 45th, and 60th, milk yield was recorded for each goat individually using hand milking in the morning and evening, and representative milk samples (50 mL) from morning and evening milking were combined to conduct milk chemical composition analysis. All the goat kids were separated from their dams for 12 h before milking. The milking was conducted by the same person to minimize the effect of human errors. The proportions (%) of milk protein, fat, lactose, total solids, in addition to somatic cell count (SCC, cells/mL) were analyzed using a MilkoScan (130 A/SN. Foss Electric, Hilleroed, Denmark).

#### Feed efficiency calculation

The gross feed efficiency (FE) was calculated as (FE) = milk production (Liter/day)/dry matter intake (DMI) (kg/day); and Fat-corrected milk for 3.5% was calculated using this formula milk yield (MY) 3.5% = (0.432 + 0.1625 × % milk fat) × milk yield, kg/day (Sklan et al. 1992). Subsequently, the adjusted feed efficiency was calculated using this equation: FE = 3.5% fat-corrected milk yield (kg)/dry matter intake (kg).

#### Microbial community

Microbial DNA was extracted from 0.5 mL of rumen fluid. Subsequently, the sample was centrifuged at 13,000 rpm for 15 min, and the pellets were used in DNA extraction using the QIAamp DNA Stool Mini Kit (Qiagen, Hilden, Germany) according to the manufacturer’s instructions. The quality and quantity of DNA were checked via gel electrophoresis and a Nanodrop spectrophotometer 2000 (Thermo Scientific, Massachusetts, United States). Rumen bacterial and archaeal communities were identified using PCR amplification of the variable V4 region on 16S rDNA. The bacterial community was investigated by amplification of the V4 region using 515F and 926R primers based on the following amplification conditions: 94 °C for 3 min; 35 cycles of 94 °C for 45 s, 50 °C for 60 s, and 72 °C for 90 s; and 72 °C for 10 min. Additionally, rumen archaea was studied by the amplification of the V4 region using primers Ar915aF (5-AGGAATTGGCGGGGGAGCAC-3) and Ar1386R (5-GCGGTGTGTGCAAGGAGC-3) (Rabee et al. 2024) based on the following PCR amplification conditions: 95 °C for 5 min; 30 cycles of 95 °C for 20 s, 55 °C for 15 s, 72 °C for 5 min, and 72 °C for 10 min. PCR amplicons were purified and sequenced using the Illumina MiSeq system (Illumina, California, United States).

#### Bioinformatics analysis

The generated paired-end raw sequence reads were analyzed using the DADA2 (version 1.11.3) pipeline through the R platform (version 3.5.2) (Callahan et al. 2016). The generated fastq files of sequence reads were demultiplexed, and their quality was checked based on the quality scores. Then, the sequences were filtered, trimmed, and dereplicated, followed by merging read 1 and read 2 together to get denoised sequences. The samples with a quality score > 30 were kept in the following analyses. The chimeras were removed from the denoised sequences to generate Amplicon Sequence Variants (ASVs).

Taxonomic assignment of ASVs was conducted using a combination of the functions assign Taxonomy and assignSpecies and was compared using of SILVA reference database (version 138). For all samples, the alpha diversity indices (observed ASVs, Chao1, Shannon, and Inverse Simpson) were calculated to analyze richness and evenness differences between the different groups; and Beta diversity was determined as principal coordinate analysis (PCoA) using Bray–Curtis dissimilarity and visualized using the phyloseq and ggplot R-packages. Function prediction of bacterial community was conducted based on the 16S rDNA data using PICRUSt2 (Douglas et al. 2020) based on the Kyoto Encyclopedia of Genes and Genomes (KEGG) database. The false discovery rate (FDR) method was used for multiple-comparison correction, and *p* values below 0.05 were considered significant. The raw sequence reads are available at https://www.ncbi.nlm.nih.gov/sra/PRJNA1141273.

### Chemical composition

The live yeast (*S. cerevisiae*, 2 * 10^7^ colony-forming units (CFU/g) is the product of the Angel yeast company (Angel yeast, Basatin, Cairo, Egypt). *Spirulina* is a commercial product of the Algal Biotechnology Unit, National Research Centre, Giza, Egypt. Dried feed and *Spirulina* samples were ground and analyzed according to the method of Association of Official Analytical Chemists (AOAC 1997) to measure dry matter (DM, method 930.15), crude protein (CP, method 954.01), ash (method 942.05), and ether extract (EE, method 920.39). Neutral detergent fiber (NDF) was measured using ANKOM Technology (ANKOM Technology, New York, United States) according to the method of Van Soest et al. (1991).

Total phenolics, total flavonoids, and total antioxidant capacity in *Spirulina* were estimated according to the method described in Elbaz et al. (2022). The antioxidant activity of *S. platensis* as 1,1-diphenyl-2-picrylhydrazyl (DPPH) was determined by the method of Herald et al. (2012). Ascorbic acid, Riboflavin, and β-Carotene were determined using high-performance liquid chromatography (HPLC) according to the method of Rahim et al. (2021). Fatty acids in *Spirulina* were determined by Gas chromatography–mass spectrometry (GC–MS) (Thermo Fisher, Waltham, MA, United States) after its conversion to fatty acid methyl esters according to Abd El-Moneim et al. (2019). Mineral content was determined according to the method of Rahim et al. (2021).

### Statistical analyses

The differences in the in vitro fermentation parameters, feed intake, milk yield and composition, feed efficiency, rumen fermentation parameters, microbial diversity, and the relative abundances of rumen bacteria and archaea were examined by the Duncan test in one-way ANOVA at *p* < 0.05. Principal component analysis (PCA), Bray–Curtis Permutational Multivariate Analysis of Variance (PERMANOVA), and Pearson correlation analyses that were visualized as a Heatmap, were conducted based on the data on the milk yield and composition, feed efficiency, rumen fermentation parameters, and the relative abundances of rumen bacteria and archaea. The statistical analyses were performed using SPSS v. 20.00 software package (SPSS 1999) and PAST (Hammer et al. 2001).

## Results

### Chemical composition of animal diets

The results showed that *Spirulina* contains 56.50% CP, 3.56% crude fiber, and 1.05% fat; in addition, *Spirulina* is a valuable source of minerals such as calcium and phosphorus, vitamins such as ascorbic and riboflavin, and phytogenic compounds such as flavonoids and phenolics (Supplementary file S1). The results revealed that *Spirulina* has higher crude protein and lower neutral detergent fiber than Alfalfa hay and CFM (Table 1; Supplementary file S1).

### In vitro dry matter disappearance and gas production

The results showed that the supplementation increased the production of gas, ammonia, and total VFA as well as dry matter digestion (DMD%) (*p* < 0.05) (Table 2). Group AY showed higher gas production and DMD, followed by groups A, C, and Y, respectively. Moreover, group AY revealed higher total VFA production, followed by groups Y, A, and C, respectively (Table 2). Higher ammonia production was observed in group A, followed by groups AY, Y, and C, respectively (Table 2). Based on these findings, the treatments AY and A were applied in the in vivo study, besides the control group (C).

**Table 2:** Effect of Spirulina and live yeast supplementation on in vitro DMD, gas production, total VFA, and ammonia–nitrogen

	Control (C)		Yeast (Y)		Spirulina (A)		Yeast + Spirulina (AY)		*p* value
	Mean	SE	Mean	SE	Mean	SE	Mean	SE	
Gas production, ml/24 h	41.33^ab^	0.66	39.00^a^	1.52	44.66^b^	0.66	56.33^c^	1.85	0.0001
Ammonia, mg/dL	24.60^a^	1.61	30.86^b^	0.46	37.40^c^	2.01	36.00^c^	2.08	0.002
VFA, meq/dL	33.30^a^	0.15	34.73^bc^	0.37	33.66^ab^	0.27	35.66^c^	0.66	0.014
DMD%	30.61^a^	0.30	35.93^b^	0.86	42.22^c^	1.74	47.67^d^	1.95	0.0001

DMD = dry matter digestion; VFA = volatile fatty acids; C = diet without supplementation; Y = diet supplemented with Saccharomyces; A = diet supplemented with Spirulina; AY = diet supplemented with saccharomyces and Spirulina

^a,b,c,d^Means within a row with different subscripts differ significantly (*p* < 0.05). SE = Standard error

### Milk yield and feed efficiency

The supplementation did not affect the feed intake (g/d) of DM, OM, EE, CP, and NDF (*p* > 0.05). While the supplementation with algae (A) and algae/yeast mixture (YA) improved average milk yield and fat-corrected milk compared to the C group (*p* < 0.05), and the difference was not significant between group A and AY. Feed efficiency expressed as gross feed efficiency and adjusted feed efficiency (Table 3) followed the same trend (*p* < 0.05). Group AY showed higher numeric fat, protein, lactose, TS, and SNF without significant difference (*p* > 0.05).

**Table 3:** Effect of *Spirulina* and live yeast supplementation on feed intake, milk yield and composition, and feed efficiency of lactating goats

	Control (C)		Spirulina (A)		Yeast + Spirulina (AY)		*p* value
	Mean	SE	Mean	SE	Mean	SE	
*Feed intake g/day*							
Animal weight, kg	34.96	1.24	36.53	1.84	33.32	1.02	0.30
DMI, g/day	1057.50	28.19	1138.66	43.07	1101.26	39.26	0.33
OMI, g/day	978.85	26.10	1053.99	39.87	1019.36	36.34	0.33
EEI, g/day	28.91	0.77	31.13	1.17	30.10	1.07	0.33
CPI, g/day	195.09	5.20	210.06	7.94	203.16	7.24	0.33
NDFI, g/day	454.98	12.13	489.90	18.53	473.81	16.89	0.33
*Milk yield (kg/day) and composition (%)*							
DMY kg/day	0.86^a^	0.07	1.17^b^	0.05	1.08^b^	0.03	0.002
Fat, %	3.33	0.22	3.70	0.19	3.89	0.23	0.21
Protein, %	2.93	0.21	2.72	0.14	3.27	0.12	0.12
Lactose, %	4.14	0.13	4.10	0.08	4.18	0.12	0.90
TS, %	11.12	0.44	10.98	0.34	12.14	0.23	0.11
SNF, %	7.48	0.32	7.51	0.21	7.96	0.11	0.24
Somatic cell count (SCC), cells/ml	397.47	8.88	342.50	24.47	375.66	16.79	0.11
*Feed efficiency (FE)*							
Gross FE	0.82^a^	0.06	1.03^b^	0.05	0.99^b^	0.05	0.03
Corrected milk, 3.5% fat	0.79^a^	0.06	1.07^b^	0.071	1.06^b^	0.07	0.01
Adjusted FE	0.83^a^	0.06	1.21^b^	0.07	1.15^b^	0.06	0.01

DMI = Dry matter intake; OMI = Organic matter intake; EEI = Ether extract intake; CPI = Crude protein intake; NDFI = Neutral detergent fiber intake; ADFI = Acid detergent fiber intake; TS = total solids; SNF = Solids Non-fats; FE = feed efficiency as Kg milk per one Kg DM^a,b,c,d^ Means within a row with different subscripts differ significantly (p < 0.05). SE = Standard error

### Rumen fermentation

Rumen pH and ammonia were similar between goat groups (*p* > 0.05) (Table 4). Goat group AY exhibited higher production of acetic acid and propionic acid, while group A showed higher butyric acid (*p* < 0.05) as well as higher numeric total VFA production (*p* > 0.05) (Table 4). Additionally, the supplementation decreased predicted methane production, and group AY showed the lowest value (*p* < 0.05) (Table 4).

**Table 4:** Effect of *Spirulina* and live yeast supplementation on rumen fermentation parameters and predicted methane of lactating goats

	Control (C)		Spirulina (A)		Yeast + Spirulina (AY)		*p* value
	Mean	SE	Mean	SE	Mean	SE	
PH	6.46	0.06	6.44	0.05	6.60	0.11	0.35
Ammonia, mg/dl	13.39	1.10	13.24	1.36	12.78	0.52	0.91
Acetic, mM	50.56^a^	2.13	50.34^a^	2.04	58.27^b^	1.44	0.02
Propionic, mM	14.77^a^	0.64	16.78^a^	1.23	18.84^b^	0.80	0.03
Isobutyric, mM	0.79	0.07	1.31	0.28	0.82	0.11	0.12
Butyric, mM	11.44^a^	0.32	14.72^b^	1.69	10.89^a^	0.49	0.047
Isovaleric, mM	0.98	0.095	2.44	0.86	1.05	0.16	0.11
Valeric, mM	2.10	0.10	2.52	0.26	2.06	0.15	0.21
Total VFA, mM	80.66	2.94	88.13	5.11	91.95	3.09	0.15
Predicted methane, g/kg DMI	25.93^b^	0.87	23.62^ab^	1.36	21.28^a^	0.69	0.02

VFA = volatile fatty acids; DMI = dry matter intake. ^a,b,c,d^ Means within a row with different subscripts differ significantly (p < 0.05). SE = Standard error

### Microbial community

#### Diversity of the bacterial community

**Fig. 1:**
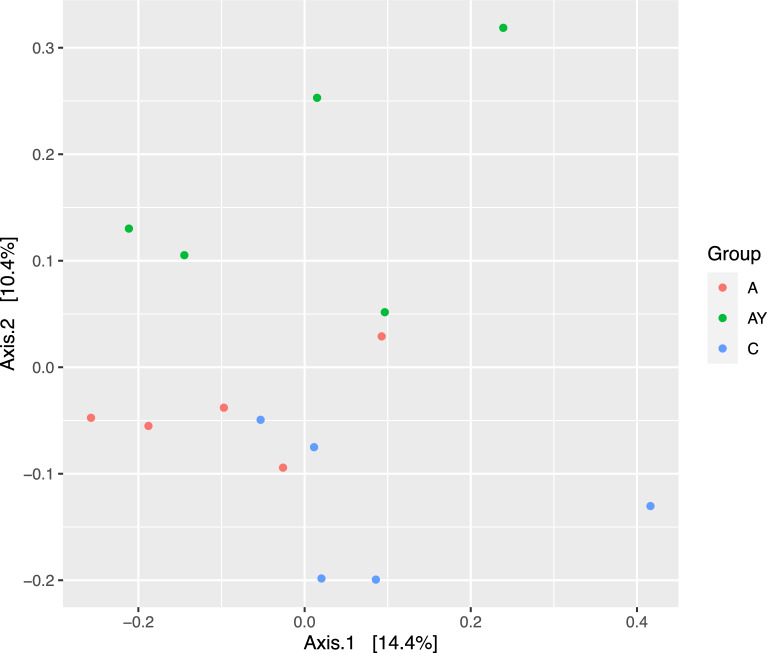
Principal coordinates analysis (PCoA) of the bacterial community was performed based on Bray–Curtis dissimilarity. The analyses were conducted between three lactating goat groups: blue circles for the control group (C), red circles for the Spirulina-supplemented group (A), and green circles for goats supplemented with a combination of *Spirulina* and yeast (AY)

The sequencing of 16S rDNA amplicons generated a total of 726,228 high-quality non-chimeric reads with a mean of 48,415 ± 5098 sequence reads per sample. The supplementation influenced alpha diversity and higher observed ASVs, Chao1, and Inverse Simpson indices were observed in the group (C), followed by groups A and AY, respectively (*p* < 0.05) (Table 5). A higher Shannon index (5.37) was observed in group C, and a lower value (4.68) was observed in group A (*p* < 0.05) (Table 5). The results PCoA of the rumen bacterial community based on Bray–Curtis dissimilarity revealed that the samples of the C group were separated from the supplemented groups (A, AY) (Fig. 1).

**Table 5:** Effect of Spirulina and live yeast supplementation on the alpha diversity indices of rumen bacteria and the relative abundances (%) of bacterial phyla in lactating goats

	Control (C)		Spirulina (A)		Yeast + Spirulina (AY)		*p* value
	Mean	SE	Mean	SE	Mean	SE	
*Alpha diversity indices*							
Observed ASVs	697.80^b^	38.05	543.20^a^	62.88	481.80^a^	11.22	0.01
Chao1	697.80^b^	38.059	543.20^a^	62.88	481.80^a^	11.22	0.01
Shannon	5.37^b^	0.04	4.68^a^	0.26	5.02^ab^	0.01	0.02
Invers Simpsone	84.95^a^	2.08	79.16^a^	3.11	64.47^b^	0.46	0.0001
*Bacterial Phyla*							
Actinobacteriota	0.04	0.014	0.10	0.03	0.08	0.02	0.24
Bacteroidota	74.29^a^	1.23	77.98^ab^	3.08	82.30^b^	0.93	0.046
Chloroflexi	0.03	0.006	0.06	0.03	0.05	0.002	0.51
Cyanobacteria	0.03	0.008	0.05	0.04	0.11	0.02	0.08
Desulfobacterota	0.29	0.09	0.16	0.07	0.41	0.19	0.39
Fibrobacterota	0.15	0.05	0.11	0.02	0.15	0.03	0.68
Firmicutes	22.27^b^	0.48	20.25^ab^	2.60	15.74^a^	0.95	0.04
Planctomycetota	0.49	0.13	0.25	0.10	0.56	0.18	0.30
Proteobacteria	0.10	0.02	0.08	0.05	0.09	0.05	0.96
Spirochaetota	0.25	0.04	0.29	0.03	0.31	0.09	0.81
Synergistota	1.19	1.07	0.08	0.06	0.13	0.0007	0.38
Verrucomicrobiota	0.25	0.04	0.21	0.08	0.37	0.14	0.55

ASVs = amplicon sequence variants. ^a,b,c,d^ Means within a row with different subscripts differ significantly (p < 0.05). SE = Standard error

#### Composition of bacterial community

Bacterial community in the rumen of goats was classified into 12 phyla, which were dominated by phylum Bacteroidota (78.19%) and Firmicutes (19.42%). Other bacterial phyla represented less than 1% of the bacterial community, including Actinobacteriota, Chloroflexi, Cyanobacteria, Desulfobacterota, Fibrobacterota, Planctomycetota, Proteobacteria, Spirochaetota, Synergistota, and Verrucomicrobiota (Table 5).

The supplementation influenced the relative abundances of Bacteroidota and Firmicutes (Table 5). Goat group AY demonstrated the highest relative abundance of phylum Bacteroidota, and the C group demonstrated the lowest value (*p* < 0.05). On the family level, the members of phylum Bacteroidota were mainly classified into families Prevotellaceae, F082, Rikenellaceae, Muribaculaceae, Bacteroidales RF16 group, and Bacteroidales BS11 gut group (Table 6). Family Prevotellaceae was dominated by the genus *Prevotella* and family Rikenellaceae was dominated by Rikenellaceae RC9 gut group*,* which showed numeric increases in AY group (*p* > 0.05) (Table 6). Phylum Firmicutes was classified mainly into Ruminococcaceae, Oscillospiraceae, Lachnospiraceae, Christensenellaceae, and Selenomonadaceae. Family Ruminococcaceae was classified mainly into genus *Ruminococcus* in addition to *Unclassified_Ruminococcaceae,* which was declined in supplemented groups (A and AY) (*p* < 0.05) (Table 6). Family Oscillospiraceae was dominated by the unclassified genus NK4A214 group and UCG-002. NK4A214 group was higher in the A group compared to the C and AY groups (*p* < 0.05). Moreover, the genus UCG-002 was increased by the supplementation (*p* < 0.05) (Table 6). Family Christensenellaceae was affiliated with the genus *Christensenellaceae R-7* group, which was increased by the supplementation (*p* < 0.05) (Table 6).

**Table 6:** Effect of Spirulina and live yeast supplementation on the relative abundances (%) of dominant rumen bacteria families and genera in lactating goats

	Control (C)		Spirulina (A)		Yeast + Spirulina (AY)		*p* value
	Mean	SE	Mean	SE	Mean	SE	
*Bacteroidota*							
F: Prevotellaceae	43.01	2.02	48.36	5.23	45.86	1.16	0.53
G: *Prevotella*	35.48	1.82	42.29	5.79	39.84	1.74	0.43
G: Prevotellaceae NK3B31 group	0.18	0.05	0.06	0.03	0.23	0.11	0.28
G: Prevotellaceae UCG-004	0.13	0.03	0.19	0.05	0.11	0.02	0.29
F: F082	15.34^a^	2.22	14.62^a^	0.70	26.74^b^	4.36	0.02
F: Rikenellaceae	8.90	0.86	10.52	0.84	11.81	2.31	0.43
G: *Rikenellaceae RC9 gut group*	8.75	0.85	10.29	0.84	11.74	2.33	0.40
F: Muribaculaceae	4.23	0.59	3.60	0.93	3.52	1.42	0.87
F: Bacteroidales RF16 group	1.97	0.37	2.18	0.32	1.39	0.56	0.44
F: Bacteroidales BS11 gut group	0.87	0.30	0.83	0.19	0.17	0.02	0.06
*Firmicutes*							
F: Ruminococcaceae	4.27	0.48	5.31	2.00	1.73	0.50	0.14
G: Ruminococcus	0.93	0.31	1.60	0.89	0.26	0.05	0.26
G: Uncu_Ruminococcaceae	3.34^b^	0.64	1.89^a^	0.15	1.63^a^	0.38	0.04
F: Oscillospiraceae	4.67	0.36	7.18	1.33	5.34	0.56	0.14
G: NK4A214 group	3.35^a^	0.43	5.83^b^	0.83	3.44^a^	0.27	0.01
G: UCG-002	0.59^a^	0.13	1.51^b^	0.18	1.80^b^	0.42	0.02
G: V9D2013 group	0.08	0.02	00	00	00	00	00
F: Christensenellaceae	3.86^a^	0.19	7.43^b^	0.78	6.34^b^	0.66	0.004
G: *Christensenellaceae R-7 group*	3.75^a^	0.21	7.40^b^	0.79	6.32^b^	0.64	0.003
F: Lachnospiraceae	2.99	0.47	2.44	0.69	4.21	0.95	0.26
G: *Butyrivibrio*	0.53	0.16	0.10	0.04	0.82	0.27	0.05
F: Selenomonadaceae	1.09	0.50	1.88	0.91	1.92	0.45	0.61
G: *Anaerovibrio*	0.05	0.02	0.21	0.13	0.21	0.06	0.30
G: *Selenomonas*	0.08	0.01	0.21	0.10	0.22	0.03	0.27
F: Anaerovoracaceae	0.22	0.02	0.20	0.03	0.19	0.05	0.85
*Spirochaetota*							
F: Spirochaetaceae; G: *Treponema*	0.10^a^	0.02	0.08^a^	0.02	0.27^b^	0.02	0.0001

F = family; G = genus. ^a,b,c,d^ Means within a row with different subscripts differ significantly (p < 0.05). SE = Standard error

#### Diversity of the archaeal community

**Fig. 2:**
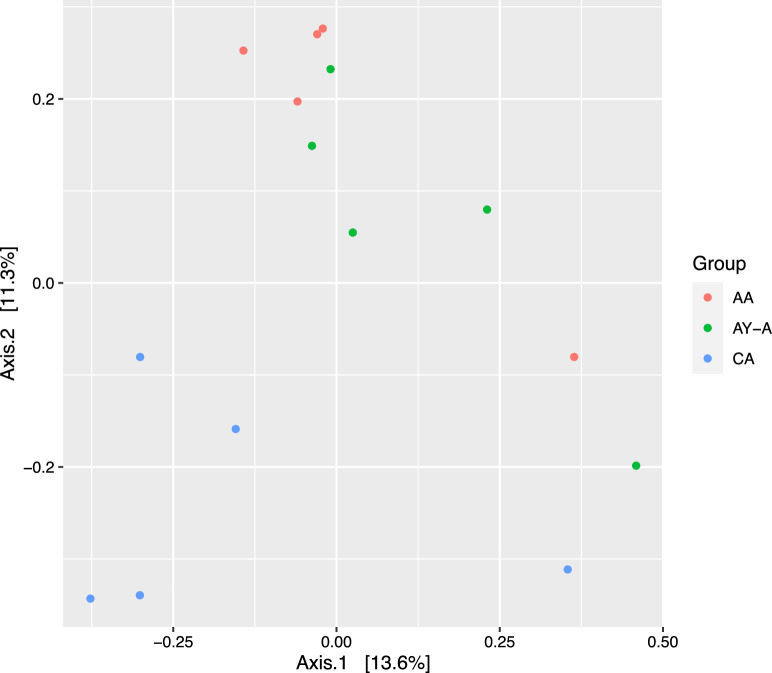
Principal coordinates analysis (PCoA) of the archaeal community was performed based on Bray–Curtis dissimilarity. The analyses were conducted between three lactating goat groups: blue circles for the archaeal community of the control group (CA), red circles for the archaeal community of the Spirulina-supplemented group (AA), and green circles for the archaeal community of goats supplemented with a combination of *Spirulina* and yeast (AY-A)

The archaeal community was analyzed in the samples of three goat groups named: C, control group; A, Spirulina-supplemented group; AY, *Spirulina* and yeast-supplemented group. The Illumina sequencing of archaeal 16S rDNA genes resulted in 264,029 sequence reads with an average of 17,601 ± 1813 sequence reads per sample. Furthermore, the sequence reads were assigned to 294 ASVs with an average of 19.6 ± 3.43 ASVs per sample. The supplementation did not influence alpha diversity indices (observed ASVs, Chao, Shannon, and Inverse Simpson) (Table 7). The results of the PCoA plot showed that archaeal communities were separated based on the dietary supplementation (Fig. 2).

**Table 7:** Effect of Spirulina and live yeast supplementation on alpha diversity indices and relative abundances (%) of rumen archaea in lactating goats

	Control (C)		Spirulina (A)		Yeast + Spirulina (AY)		*p* value
	Mean	SE	Mean	SE	Mean	SE	
Observed ASVs	29.00	6.97	20.60	4.10	25.60	4.40	0.54
Chao1	29.00	6.97	20.60	4.10	25.60	4.40	0.54
Shannon	2.18	0.54	2.28	0.14	2.71	0.16	0.51
Invers Simpsone	8.83	3.18	6.98	0.82	11.99	1.71	0.28
Euryarchaeota; Methanobacteria; Methanobacteriales; Methanobacteriaceae							
*Methanobrevibacter*	75.59^b^	1.76	78.06^b^	1.45	53.34^a^	4.61	0.0001
*Methanosphaera*	00	00	0.16	0.02	0.33	0.29	00
*Methanobacterium*	00	00	0.14	0.04	00	00	00
Thermoplasmatota; Thermoplasmata; Methanomassiliicoccales; Methanomethylophilaceae;							
Candidatus Methanomethylophilus	13.11	0.46	7.40	0.43	11.68	2.81	0.07
Methanomethylophilaceae _Unclassif	11.23^a^	1.26	13.40^a^	0.95	34.15^b^	7.22	0.004
Halobacterota; Methanosarcinia; Methanosarciniales; Methanosarcinaceae;							
*Methanimicrococcus*	0	0	0.71	0.30	0.38	0.18	00

ASV = amplicon sequence variants. ^a,b,c,d^ Means within a row with different subscripts differ significantly (p < 0.05). SE = Standard error

### *Composition of archaeal community*

The ruminal archaeal community was classified into three phyla, Euryarchaeota, Thermoplasmatota, and Halobacterota, which were affected by the supplementation (Table 7). The members of phylum Euryarchaeota belonged to the family Methanobacteriaceae, which were classified into three genera: *Methanobrevibacter*, *Methanosphaera*, and *Methanobacterium* (Table 7). Genus *Methanobrevibacter* dominated the archaeal community and was declined in group AY compared to groups C and A (*p* < 0.05) (Table 7). Genus *Methanosphaera* was detected only in groups A and AY, and genus *Methanobacterium* was detected only in group A (Table 7). The members of phylum Thermoplasmatota belonged to the family Methanomethylophilaceae, which was further classified as *Candidatus Methanomethylophilus* and *Unclassified_Methanomethylophilaceae* that showed their higher relative abundances (*p* < 0.05) in group AY compared to groups C and A. Phylum Halobacterota was classified into family Methanosarcinaceae and genus *Methanimicrococcus* that was detected only in group A and AY.

### Principal component analysis (PCA) and Bray–Curtis permutational multivariate analysis of variance (PERMANOVA)

**Fig. 3:**
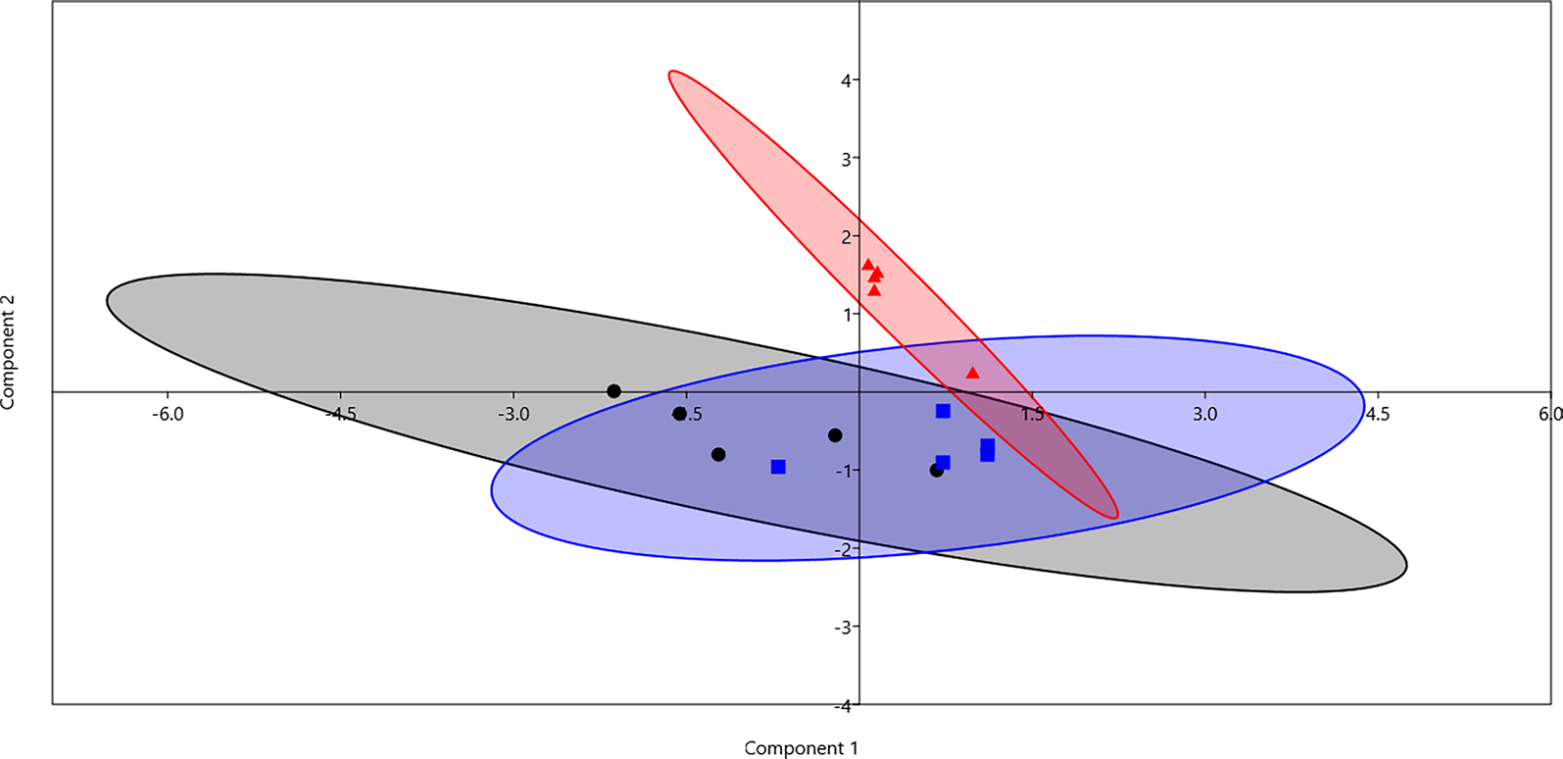
Principal component analysis (PCA) was determined using the results of milk yield and composition, feed efficiency, rumen fermentation parameters, and the relative abundances of dominant bacteria and archaea. The black dots are for the control group (C), the blue squares are for group A, and the red triangles are for group AY

PCA analysis was performed using the data of milk yield and composition, feed efficiency, rumen fermentation parameters, and the relative abundances of dominant bacteria and archaea (Fig. 3). The results revealed that the samples were separated based on the dietary treatments, and this result was supported by a significant difference (*p* = 0.036) obtained by the PERMANOVA test. The separation in PCA was driven by milk yield, total VFA, and the relative abundance of phyla Bacteroidota and Firmicutes as well as genus *Methanobrevibacter.*

### Correlation analysis

Pearson correlation analysis was conducted between milk yield and composition, feed efficiency, rumen fermentation parameters, and the relative abundances of dominant bacteria and archaea. The correlation relationships were visualized in the heatmap (Supplementary Figure S1). The heatmap showed several positive and negative correlation relationships. Milk yield correlated positively with feed efficiency, rumen ammonia, phylum Bacteroidota, Rikenellaceae RC9 gut group, and *Christensenellaceae R-7 group*. Adjusted feed efficiency correlated positively with total VFA, propionic acid, and phylum Bacteroidota. There was a negative correlation between the feed efficiency and the relative abundances of Fibrobacterota and Firmicutes. A negative correlation was observed between feed efficiency and genus *Methanobrevibacter*.

### Function prediction of rumen bacteria

PICRUSt2 functional prediction was conducted using the relative abundance of metabolic pathways and was visualized as Principal Components Analysis (PCA) (Supplementary figure S2). A slight separation of samples was noted based on the dietary supplementation. Supplementation with *Spirulina* in group (A) enriched the rumen bacteria in Flavon and Flavonol metabolites, whereas group (AY) that received a combination of yeast and *Spirulina* was enriched in D-glutamine and Glutamate metabolites.

## Discussion

Feed supplements containing microalgae such as *Spirulina platensis* and *Saccharomyces cerevisiae* are effective in improving animal productivity. *Spirulina* provides several nutrients and bioactive compounds (Supplementary file S1) that could enhance the performance of *Saccharomyces*, rumen microbiota, and host animal (Rabee et al. 2022; El-Deeb et al. 2023). *Saccharomyces* optimizes the rumen anaerobic conditions and stabilizes rumen pH, which enhances cellulolytic microorganisms and fiber digestion (Rabee et al. 2022). These explanations justify the changes in bacterial and archaeal communities, and enhancements in rumen fermentation, milk yield, and feed efficiency.

The diets supplemented with *Spirulina* (A) or a mixture of *Spirulina* and yeast (AY), had higher in vitro rumen fermentation parameters (Table 2). A similar trend was obtained by when soybean meal was replaced by *Spirulina* in the dairy cattle diet (Lobo et al. 2024). Microalgae are rich in carbohydrates that are converted to VFAs during the microbial fermentation (Lobo et al. 2024). Moreover, inclusion of live yeast in animal diets improved diet digestion and VFA production (Phesatcha et al. 2020). Higher gas production is associated with higher microbial growth and substrate fermentation (Getachew et al. 1998).

The Goat supplemented with a combination of *Spirulina* and live yeast (AY) had higher total VFA, acetic and propionic acids (Table 4). Similar findings were observed in camel and sheep supplemented with a combination of live yeast and microalgae mixture (Rabee et al. 2022). Live yeast and microalgae modulate rumen microbial communities to enhance rumen fermentation (Lobo et al. 2024; Zhang et al. 2023). This explanation is supported by higher acetic production, which refers to stimulated microbial fiber degradation in rumen (Getachew et al. 1998). Higher butyrate is a positive point of *Spirulina* supplementation in group A as butyrate maintains the ruminal papillae and enhances the absorption of nutrients from the rumen (O'Shea et al. 2016). Higher propionic production declines methane production as propionic-producing bacteria consume hydrogen molecules to synthesize propionic (Meehan et al. 2021). Hydrogen is the main substrate that methanogens use to produce methane which explains the lower predicted methane in supplemented groups (A and AY) (Rabee et al. 2024). The decline in the methane production improves animal performance as methane production represents a loss in the host's gross energy (Rabee et al. 2024).

The changes in rumen fermentation were associated with changes in rumen bacteria and archaea due to the supplementation. The decline in bacterial diversity indices in supplemented groups (A and AY) (Table 5; Fig. 1), was previously reported in sheep supplemented with *Spirulina* (Wang et al. 2023). Brooke et al. (2019) found that lower rumen bacterial diversity was linked with higher feed efficiency, which highlights the current findings. Phyla Bacteroidota and Firmicutes dominated the bacterial community (Table 5), which agrees with previous study on goats supplemented with phytochemicals (Rabee et al. 2024). The members of phylum Bacteriodota utilize a wide range of substrates such as cellulose, pectin, and soluble polysaccharides and were increased due to phytochemical supplementation (Rabee et al. 2024). *Spirulina* (group A and AY) is known as a rich source of phenols and flavonoids (Elbaz et al. 2022), which supports the current findings.

Genus *Prevotella* utilizes a wide range of substrates such as hemicellulose and protein (Matsui et al. 2000). Moreover, this genus is the main propionate producer in the rumen (De Vadder et al. 2016), which explains the higher propionic in supplemented groups due to the increase of the prevalence of *Prevotella* as a result of supplementation. The increase of *Prevotella* and propionate is another explanation of declined methane production in supplemented groups (Betancur-Murillo et al. 2022). Genus Rikenellaceae RC9 gut group*,* within phylum Bacteriodota, can utilize dietary fiber and produces acetic and propionic, which highlight higher acetic and propionic in supplemented groups (A and AY) (Rabee et al. 2024). Moreover, family F082 was a higher group AY; this family has a potential role in the degradation of soluble carbohydrates (Yi et al. 2022). These findings were confirmed by the positive correlation between milk yield, feed efficiency, VFA production, propionic acid, phylum Bacteroidota, and genus Rikenellaceae RC9 gut group.

Phylum Firmicutes was dominated by the family Ruminococcaceae and Christensenellaceae (Table 6), which agrees with Rabee et al. (2022). Genus *Christensenellaceae R-7 group,* within family Christensenellaceae was higher in group A and AY. This genus has a potential role in fiber and protein digestion and produces acetic and butyric acids; besides, it was associated with higher feed efficiency (Bach et al. 2019; Huang et al. 2021), which highlight the positive effect of the supplementation.

*Spirulina* supplementation in group A increased the metabolic pathways related to Flavon and Flavonol. Flavon and Flavanol are types of flavonoids that are enriched in *Spirulina* (Elbaz et al. 2022) and have antioxidant, antimicrobial, immunostimulant, and anti-inflammatory activities (Bach et al. 2019; Huang et al. 2021). A higher relative abundance of pathways related to flavonoids in group A refers to higher availability and utilization of flavonoids and higher microbial species that utilize flavonoids (Hostetler et al. 2017; Zhou et al. 2023), which deliver several beneficial effects to host animals and improve their performance. Group AY, fed a combination of yeast and *Spirulina*, was enriched in D-Glutamine and Glutamate metabolism. Rumen bacteria use glutamine to build microbial protein that provides the host animal with protein; therefore, this amino acid is vital in animal health and productivity (Wu et al. 2024). Furthermore, glutamine is essential for nitrogen balance in the rumen as glutamine is hydrolyzed by the rumen mucosal homogenate to generate ammonia; and rumen ammonia is converted to glutamine at a high ammonia level, which reduces the loss of ammonia in the rumen (Hoshino et al. 1966). Also, glutamine is essential to the proliferation of immune cells, which supports animal immunity and performance (Lobley et al. 2001). A higher relative abundance of glutamine-related pathways in the AY group highlights the yeast-Spirulina supplementation to improve animal health and productivity, especially under heat stress conditions that reduce glutamine availability in ruminant animals (Li et al. 2022).

The composition and abundance of rumen archaea are affected mainly by diet composition, which affects the fermentation type and available growth substrates (Rabee et al. 2024). This finding explains the decline of genus *Methanobrevibacter* in the AY group. *Methanobrevibacter* produces methane using hydrogen, acetate, and formate, which leads to energy losses (Jeyanathan et al. 2011). This genus is the main methane producer in the rumen and was associated with higher methane production and low feed efficiency in steers (Bharanidharan et al. 2018). This finding was supported by the negative correlation between *Methanobrevibacter* and feed efficiency (Supplementary figure S1). Another explanation for the decline of the *Methanobrevibacter* in the AY group is the presence of phenol compounds in *Spirulina*; these compounds have negative impacts on rumen methanogens (Sucu 2023; Rabee et al. 2024). Furthermore, Wang et al. (2023) indicated that polyunsaturated fatty acids in *Spirulina* decline the rumen methanogens by declining the available hydrogen and increasing production of propionic acid. Members of the family Methanomethylophilaceae drive energy from the metabolism of methanol and methylamine and were associated with lower methane emission and higher feed efficiency in sheep (Borrel et al. 2012; McLoughlin et al. 2023), which highlights the increase of this family in AY group.

In this study, the improvement in milk yield due to yeast and *Spirulina* (Table 3) was also indicated in previous studies on sheep (El-Deeb et al. 2023) and cows (Kulpys et al. 2009; Tristant and Moran 2015). However, no studies investigated a combination of live yeast and *Spirulina* on milk yield. In this study, higher milk yield in the supplemented group could be attributed to a higher production of VFA and microbial protein and the decline in methane emission, which increases the energy and protein supply to the host animal (Rabee et al. 2022; El-Deeb et al. 2023). This finding was confirmed by the positive correlation (Supplementary figure S1) between milk yield, feed efficiency, VFA, and propionic acid. Bioactive compounds were reported to improve animal health by improving blood immunity and antioxidant capacity, which increases feed conversion (El-Deeb et al. 2023). Kholif et al. (2020) attributed the improvement in milk yield of goats supplemented with microalgae (*Nannochloropsis*) to the improvements in the digestibility of diets and VFA production. Propionic acid is a precursor for gluconeogenesis and lactose synthesis, which improves milk yield (Rigout et al. 2003). Interestingly, the supplementation with *Spirulina* alone resulted in higher gas production but similar VFA production to that of *Saccharomyces* alone, confirming a higher influence of *Spirulina* on fiber utilization (Rabee et al. 2022; Calabrò et al. 2005). Tristant and Moran (2015) reported that yeast declines lactic acid production, which enhances microbial fermentation, diet digestibility, and microbial protein production.

Despite the promising findings of this study, it is highly recommended to repeat this study on the commercial sector with a larger sample size in each group.

The yeast and algae supplementation (group A and AY) in goats increased fiber-degrading bacteria and decreased the major rumen archaea, which increased the total VFA production and decreased methane production. Consequently, milk yield and feed efficiency were enhanced. Therefore, these findings provide a rationale for the inclusion of a combination of yeast and *Spirulina* at 1% of DM intake in the diet of lactating goats. Future studies are recommended to optimize the inclusion levels of Spirulina-yeast combinations to enhance ruminant productivity and rumen function.

## Supplementary Information

Supplementary Material 1.

Supplementary Material 2.

Supplementary Material 3.

Supplementary Material 4.

## Data Availability

The raw sequence reads are available at https://www.ncbi.nlm.nih.gov/sra/PRJNA1141273.

## Abbreviations

ASVs: Amplicon sequence variants
PERMANOVA: Permutational multivariate analysis of variance
DPPH: 1,1-Diphenyl-2-picrylhydrazyl
DMD: Dry matter digestibility
FDR: False discovery rate
FE: Feed efficiency
HPLC: High-performance liquid chromatographic
KEGG: Kyoto Encyclopedia of Genes and Genomes database.
MY: Milk yield
PCA: Principal component analysis
PCoA: Principal coordinate analysis
SCC: Somatic cell count
VFA: Volatile fatty acids (VFA)

## References

[CR1] Abd El-Moneim AE, Sabic EM, Abu-Taleb AM (2019) Influence of dietary supplementation of irradiated or non-irradiated olive pulp on biochemical profile, antioxidant status and immune response of Japanese quails. Biol Rhythm Res 53(4):519–534. 10.1080/09291016.2019.1630919

[CR2] Almasri O, Abou-Bakr S, Ibrahim M, Kahil O, Asaad Z, Ghoush M, Awad M (2023) Lactation curve and milk production traits of Syrian Damascus goats. Egypt J Anim Prod 60(1):7–16. 10.21608/ejap.2023.177189.1051

[CR3] Alsheikh SM (2013) Influence of age and live body weight on daily milk yield of Zaraibi and Shami goats in Sinai. Egypt. Ann Agric Sci 58:1–3. 10.1016/j.aoas.2013.01.001

[CR4] AOAC (1997) Association of Official Analytical Chemists international official methods of analysis, 16th edn. AOAC, Arlington

[CR5] Bach A, López-García A, González-Recio O, Elcoso G, Fàbregas F, Chaucheyras-Durand F, Castex M (2019) Changes in the rumen and colon microbiota and effects of live yeast dietary supplementation during the transition from the dry period to lactation of dairy cows. J Dairy Sci 102(7):6180–6198. 10.3168/jds.2018-16105 31056321 10.3168/jds.2018-16105

[CR6] Betancur-Murillo CL, Aguilar-Marín SB, Jovel J (2022) *Prevotella*: a key player in ruminal metabolism. Microorganisms 11(1):1–18. 10.3390/microorganisms11010001 36677293 10.3390/microorganisms11010001 PMC9866204

[CR7] Bharanidharan R, Arokiyaraj S, Kim EB, Lee CH, Woo YW, Na Y, Kim D, Kim KH (2018) Ruminal methane emissions, metabolic, and microbial profile of Holstein steers fed forage and concentrate, separately or as a total mixed ration. PLoS ONE 13(8):e0202446. 10.1371/journal.pone.0202446 30110381 10.1371/journal.pone.0202446 PMC6093700

[CR8] Borrel G, Harris HM, Tottey W, Mihajlovski A, Parisot N, Peyretaillade E, Peyret P, Gribaldo S, O’Toole PW, Brugère JF (2012) Genome sequence of “*Candidatus Methanomethylophilusalvus*” Mx1201, a methanogenic archaeon from the human gut belonging to a seventh order of methanogens. J Bacteriol 194(24):6944–6945. 10.1128/JB.01867-12 23209209 10.1128/JB.01867-12 PMC3510639

[CR9] Brooke CG, Najafi N, Dykier KC, Hess M (2019) *Prevotella copri*, a potential indicator for high feed efficiency in western steers. Anim Sci J 90(5):696–701. 10.1111/asj.13197 30848016 10.1111/asj.13197

[CR10] Calabrò S, Cutrignelli MI, Bovera F, Piccolo G, Infascelli F (2005) *In vitro* fermentation kinetics of carbohydrate fractions of fresh forage, silage and hay of Avena sativa. J Sci Food Agric 85:1838–1844. 10.1002/jsfa.2186

[CR11] Callahan BJ, McMurdie PJ, Rosen MJ, Han AW, Johnson AJ, Holmes SP (2016) DADA2: high-resolution sample inference from Illumina amplicon data. Nat Methods 13(7):581–583. 10.1038/nmeth.3869 27214047 10.1038/nmeth.3869 PMC4927377

[CR12] Christodoulou C, Mavrommatis A, Loukovitis D, Symeon G, Dotas V, Kotsampasi B, Tsiplakou E (2023) Effect of *Spirulina* dietary supplementation in modifying the rumen microbiota of Ewes. Animals 13:740. 10.3390/ani13040740 36830527 10.3390/ani13040740 PMC9952741

[CR13] De Vadder F, Kovatcheva-Datchary P, Zitoun C, Duchampt A, Bäckhed F, Mithieux G (2016) Microbiota-produced succinate improves glucose homeostasis via intestinal gluconeogenesis. Cell Metab 24(1):151–157. 10.1016/j.cmet.2016.06.013 27411015 10.1016/j.cmet.2016.06.013

[CR14] Douglas GM, Maffei VJ, Zaneveld JR, Yurgel SN, Brown JR, Taylor CM, Huttenhower C, Langille MGI (2020) PICRUSt2 for prediction of metagenome functions. Nat Biotechnol 38(6):685–688. 10.1038/s41587-020-0548-6 32483366 10.1038/s41587-020-0548-6 PMC7365738

[CR15] Elbaz AM, Ahmed AMH, Abdel-Maqsoud A, Badran AMM, Abdel-Moneim AE (2022) Potential ameliorative role of *Spirulina platensis* in powdered or extract forms against cyclic heat stress in broiler chickens. Environ Sci Pollut Res Int 29(30):45578–45588. 10.1007/s11356-022-19115-z 35149947 10.1007/s11356-022-19115-z PMC9209341

[CR16] El-Deeb MM, Abdel-Gawad M, Abdel-Hafez MAM, Saba FE, Ibrahim EMM (2023) Effect of adding *Spirulina platensis* algae to small ruminant rations on productive, reproductive traits and some blood components. Acta Sci Anim Sci 45:e57546. 10.4025/actascianimsci.v45i1.57546

[CR17] Finamore A, Palmery M, Bensehaila S, Peluso I (2017) Antioxidant, immunomodulating, and microbial-modulating activities of the sustainable and ecofriendly *Spirulina*. Oxid Med Cell Longev 2017:3247528. 10.1155/2017/3247528 28182098 10.1155/2017/3247528 PMC5274660

[CR18] Getachew G, Blümmel M, Makkar HPS, Becker K (1998) *In vitro* gas measuring techniques for assessment of nutritional quality of feeds: a review. Anim Feed Sci Technol 72:261–281. 10.1016/S0377-8401(97)00189-2

[CR19] Grimm P, Combes S, Pascal G, Cauquil L, Julliand V (2020) Dietary composition and yeast/microalgae combination supplementation modulate the microbial ecosystem in the caecum, colon and faeces of horses. Br J Nutr 123(4):372–382. 10.1017/S0007114519002824 31690358 10.1017/S0007114519002824

[CR20] Gupta S, Gupta C, Prakash D (2017) Prebiotic efficiency of blue green algae on probiotics microorganisms. J Microbiol Exp 4(4):00120. 10.15406/jmen.2017.04.00120

[CR21] Hadhoud F, Abd El Tawab A, Khattab M (2022) Benefits of supplementing yeast to diets on dairy animals’ performance. Egypt J Chem 65(8):109–124. 10.21608/ejchem.2022.103649.4795

[CR22] Hammer Ø, Harper DAT, Ryan PD (2001) PAST: paleontological statistics software package for education and data analysis. Palaeontol Electron 4:9

[CR23] Herald TJ, Gadgil P, Tilley M (2012) High-throughput microplate assays for screening flavonoid content and DPPH-scavenging activity in sorghum bran and flour. J Sci Food Agric 92(11):2326–2331. 10.1002/jsfa.5633 22419130 10.1002/jsfa.5633

[CR24] Hoshino S, Sarumaru K, Morimoto K (1966) Ammonia anabolism in ruminants. J Dairy Sci 49(12):1523–1528

[CR25] Hostetler GL, Ralston RA, Schwartz SJ (2017) Flavones: food sources, bioavailability, metabolism, and bioactivity. Adv Nutr 8(3):423–435. 10.3945/an.116.012948 28507008 10.3945/an.116.012948 PMC5421117

[CR26] Huang C, Ge F, Yao X, Guo X, Bao P, Ma X, Wu X, Chu M, Yan P, Liang C (2021) Microbiome and metabolomics reveal the effects of different feeding systems on the growth and ruminal development of Yaks. Front Microbiol 12:682989. 10.3389/fmicb.2021.682989 34248900 10.3389/fmicb.2021.682989 PMC8265505

[CR27] Jeyanathan J, Kirs M, Ronimus RS, Hoskin SO, Janssen PH (2011) Methanogen community structure in the rumens of farmed sheep, cattle and red deer fed different diets. FEMS Microbiol Ecol 76(2):311–326. 10.1111/j.1574-6941.2011.01056.x 21255054 10.1111/j.1574-6941.2011.01056.x

[CR28] Kholif AE, Gouda GA, Hamdon HA (2020) Performance and milk composition of Nubian goats as affected by increasing level of *Nannochloropsis oculata* microalgae. Animals 10(12):2453. 10.3390/ani10122453 33371450 10.3390/ani10122453 PMC7767434

[CR29] Kulpys J, Paulauskas E, Pilipavičius V, Stankevičius R (2009) Influence of *cyanobacteria Arthrospira* (Spirulina) platensis biomass additives towards the body condition of lactation cows and biochemical milk indexes. Agron Res 7:823–835

[CR30] Li C, Zhang J, Li Y, Zhao X, Liang H, Li K, Qu M, Qiu Q, Ouyang K (2022) Glutamate supplementation improves growth performance, rumen fermentation, and serum metabolites in heat-stressed Hu sheep. Front Nutr 9:851386. 10.3389/fnut.2022.851386 35464012 10.3389/fnut.2022.851386 PMC9026332

[CR31] Lobley GE, Hoskin SO, McNeil CJ (2001) Glutamine in animal science and production. J Nutr 131(9 Suppl):2525S-2531S. 10.1093/jn/131.9.2525S. (**discussion 2532S-4S**) 11533306 10.1093/jn/131.9.2525S

[CR32] Lobo RR, Siregar MU, da Silva SS, Monteiro AR, Salas-Solis G, Vicente ACS, Vinyard JR, Johnson ML, Ma S, Sarmikasoglou E, Coronella CJ, Hiibel SR, Faciola AP (2024) Partial replacement of soybean meal with microalgae biomass on *in vitro* ruminal fermentation may reduce ruminal protein degradation. J Dairy Sci 107(3):1460–1471. 10.3168/jds.2023-24016 37944802 10.3168/jds.2023-24016

[CR33] Markowiak P, Śliżewska K (2018) The role of probiotics, prebiotics and synbiotics in animal nutrition. Gut Pathog 10:21. 10.1186/s13099-018-0250-0 29930711 10.1186/s13099-018-0250-0 PMC5989473

[CR34] Mašek T, Mikulec Ž, Valpotić H, Antunac N, Mikulec N, Stojević Z, Filipović N, Pahović S (2008) Influence of live yeast culture (*Saccharomyces cerevisiae*) on milk production and composition, and blood biochemistry of grazing dairy ewes during the milking period. Acta Vet Brno 77:547–554. 10.2754/avb200877040547

[CR35] Matsui H, Ogata K, Tajima K, Nakamura M, Nagamine T, Aminov RI, Benno Y (2000) Phenotypic characterization of polysaccharides produced by four *Prevotella* type strains. Curr Microbiol 41(1):45–49. 10.1007/s002840010089 10919398 10.1007/s002840010089

[CR36] McLoughlin S, Spillane C, Campion FP, Claffey N, Sosa CC, McNicholas Y, Paul S, Michael D, Sinéad W (2023) Breed and ruminal fraction effects on bacterial and archaeal community composition in sheep. Sci Rep 3(1):3336. 10.1038/s41598-023-28909-1 10.1038/s41598-023-28909-1 PMC9971215 36849493

[CR37] Meehan D, Cabrita ARJ, Silva JL, Fonseca AJM, Maia MRG (2021) Effects of *Chlorella vulgaris*, *Nannochloropsis oceanica* and *Tetraselmis sp*. supplementation levels on *in vitro* rumen fermentation. Algal Res 56:102284. 10.1016/j.algal.2021.102284

[CR38] Menke KH, Raab L, Salewski A, Steingass H, Fritz D, Schneider W (1979) The estimation of the digestibility and metabolizable energy content of ruminant feeding stuffs from the gas production when they are incubated with rumen liquor in vitro. J Agric Sci 93(1):217–222. 10.1017/S0021859600086305

[CR39] National Research Council, NRC (2007) Nutrient requirements of small ruminants: sheep, goats, cervids, and New World camelids. The National Academies Press, Washington DC

[CR40] O’Shea E, Waters SM, Keogh K, Kelly AK, Kenny DA (2016) Examination of the molecular control of ruminal epithelial function in response to dietary restriction and subsequent compensatory growth in cattle. J Anim Sci Biotechnol 7:53. 10.1186/s40104-016-0114-8 27651894 10.1186/s40104-016-0114-8 PMC5025635

[CR41] Phesatcha K, Phesatcha B, Wanapat M, Cherdthong A (2020) Roughage to concentrate ratio and *Saccharomyces cerevisiae* inclusion could modulate feed digestion and *in vitro* ruminal fermentation. Vet Sci 7(4):151. 10.3390/vetsci7040151 33050260 10.3390/vetsci7040151 PMC7712883

[CR42] Rabee AE, Younan BR, Kewan KZ, Sabra EA, Lamara M (2022) Modulation of rumen bacterial community and feed utilization in camel and sheep using combined supplementation of live yeast and microalgae. Sci Rep 12(1):12990. 10.1038/s41598-022-16988-5 35906456 10.1038/s41598-022-16988-5 PMC9338284

[CR43] Rabee AE, Ghandour MMM, Sallam A, Elwakeel EA, Mohammed RS, Sabra EA, Abdel-Wahed AM, Mourad DM, Hamed AA, Hafez OR (2024) Rumen fermentation and microbiota in Shami goats fed on condensed tannins or herbal mixture. BMC Vet Res 20(1):35. 10.1186/s12917-024-03887-2 38297287 10.1186/s12917-024-03887-2 PMC10829277

[CR44] Rahim A, Çakir C, Ozturk M, Şahin B, Soulaimani A, Sibaoueih M, Nasser B, Eddoha R, Essamadi A, El Amiri B (2021) Chemical characterization and nutritional value of *Spirulina platensis* cultivated in natural conditions of Chichaoua region (Morocco). S Afr J Bot 141:235–242

[CR45] Rigout S, Hurtaud C, Lemosquet S, Bach A, Rulquin H (2003) Lactational effect of propionic acid and duodenal glucose in cows. J Dairy Sci 86(1):243–253. 10.3168/jds.S0022-0302(03)73603-0 12613868 10.3168/jds.S0022-0302(03)73603-0

[CR46] Sklan D, Ashkenazi R, Braun A, Devorin A, Tabori K (1992) Fatty acids, calcium soaps of fatty acids, and cottonseeds fed to high yielding cows. J Dairy Sci 75(9):2463–2472. 10.3168/jds.S0022-0302(92)78008-4 1452851 10.3168/jds.S0022-0302(92)78008-4

[CR47] SPSS (1999) Statistical package for social science Release 15. SPSS INC, Chicago, USA

[CR48] Sucu E (2023) *In Vitro* studies on rumen fermentation and methanogenesis of different microalgae and their effects on acidosis in dairy cows. Fermentation 9:229. 10.3390/fermentation9030229

[CR49] Tristant D, Moran CA (2015) The efficacy of feeding a live probiotic yeast, Yea-Sacc®, on the performance of lactating dairy cows. J Appl Anim Nutr 3:e12. 10.1017/jan.2015.10

[CR50] Van Soest PJ, Robertson JB, Lewis BA (1991) Methods for dietary fiber, neutral detergent fiber, and nonstarch polysaccharides in relation to animal nutrition. J Dairy Sci 74(10):3583–3597. 10.3168/jds.S0022-0302(91)78551-2 1660498 10.3168/jds.S0022-0302(91)78551-2

[CR51] Wang Z, Liang Y, Lu J, Wei Z, Bao Y, Yao X, Fan Y, Wang F, Wang D, Zhang Y (2023) Dietary spirulina supplementation modifies rumen development, fermentation and bacteria composition in Hu sheep when consuming high-fat dietary. Front Vet Sci 10:1001621. 10.3389/fvets.2023.1001621 36798143 10.3389/fvets.2023.1001621 PMC9926970

[CR52] Williams SRO, Hannah MC, Jacobs JL, Wales WJ, Moate PJ (2019) Volatile fatty acids in ruminal fluid can be used to predict methane yield of dairy cows. Animals 9(12):1006. 10.3390/ani9121006 31757116 10.3390/ani9121006 PMC6941164

[CR53] Wu G, Bazer FW, Johnson GA, Satterfield MC, Washburn SE (2024) Metabolism and nutrition of L-glutamate and L-glutamine in ruminants. Animals 14(12):1788. 10.3390/ani14121788 38929408 10.3390/ani14121788 PMC11201166

[CR54] Xue MY, Xie YY, Zhong Y, Ma XJ, Sun HZ, Liu JX (2022) Integrated meta-omics reveals new ruminal microbial features associated with feed efficiency in dairy cattle. Microbiome 10(1):32. 10.1186/s40168-022-01228-9 35172905 10.1186/s40168-022-01228-9 PMC8849036

[CR55] Yi S, Dai D, Wu H, Chai S, Liu S, Meng Q, Zhou Z (2022) Dietary concentrate-to-forage ratio affects rumen bacterial community composition and metabolome of Yaks. Front Nutr 9:927206. 10.3389/fnut.2022.927206 35911107 10.3389/fnut.2022.927206 PMC9329686

[CR56] Zhang J, Yang Y, Lei X, Wang Y, Li Y, Yang Z, Yao J (2023) Active dry yeast supplementation benefits ruminal fermentation, bacterial community, blood immunoglobulins, and growth performance in young dairy goats, but not for intermittent supplementation. Anim Nutr 13:289–301. 10.1016/j.aninu.2023.02.001 37168451 10.1016/j.aninu.2023.02.001 PMC10165222

[CR57] Zhou M, Ma J, Kang M, Tang W, Xia S, Yin J, Yin Y (2023) Flavonoids, gut microbiota, and host lipid metabolism. Eng Life Sci 24(5):2300065. 10.1002/elsc.202300065 38708419 10.1002/elsc.202300065 PMC11065335

