# Supplementary material for: Milk yield, rumen fermentation, and microbiota of Shami goats fed diets supplemented with spirulina and yeast
Source: AMB Express. 2025 Jul 21;15:108. doi: 10.1186/s13568-025-01916-3 (PMC12279650; doi:10.1186/s13568-025-01916-3)
Supplement: Supplementary file 1 — Supplementary Material 1. [file 13568_2025_1916_MOESM1_ESM.pdf]

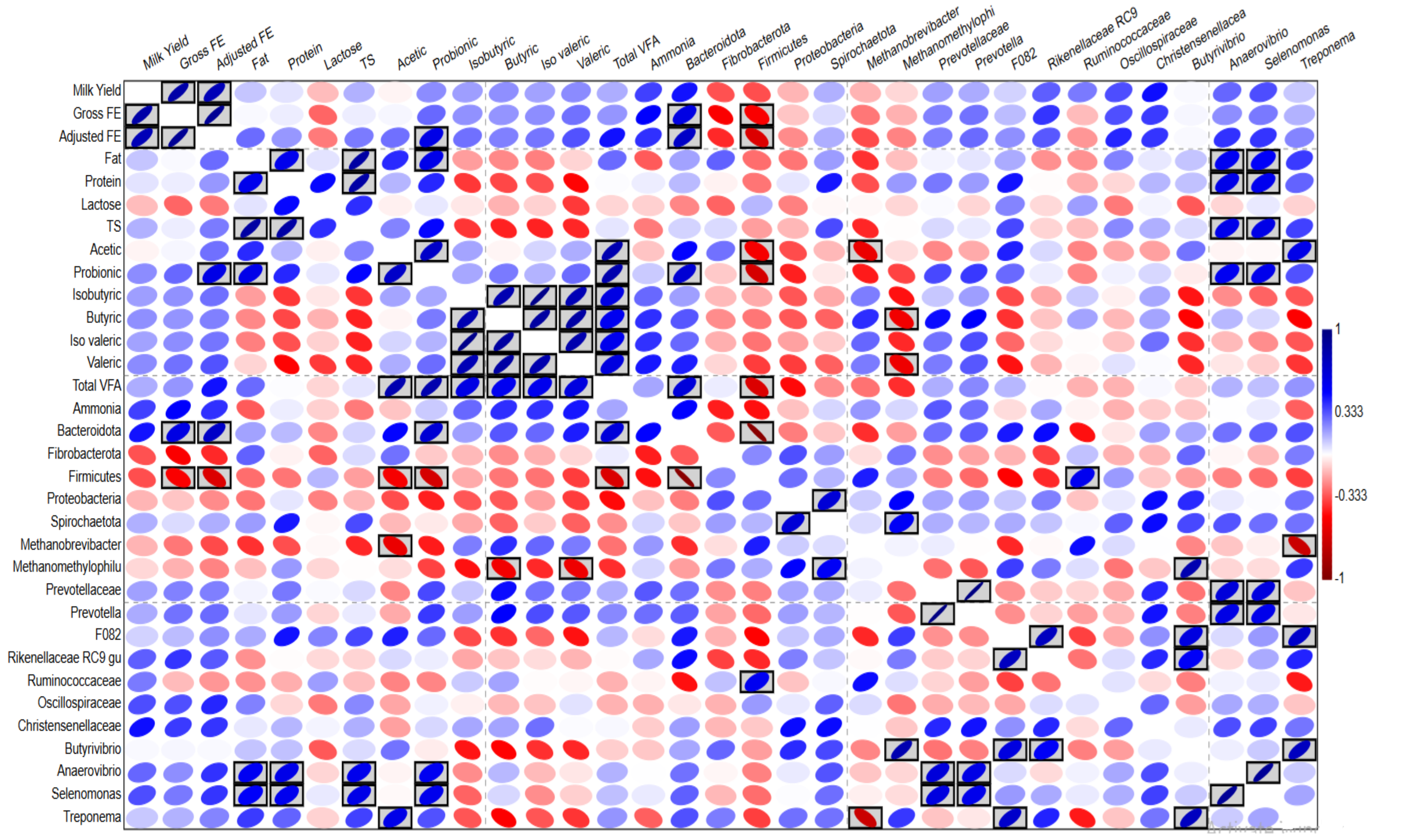

Supplementary figure S1: Pearson correlation analysis visualized as a heatmap. The correlation relationships were conducted between milk yield and composition, feed efficiency, rumen fermentation parameters, and the relative abundances of dominant bacteria and archaea.
