# Supplementary material for: Milk yield, rumen fermentation, and microbiota of Shami goats fed diets supplemented with spirulina and yeast
Source: AMB Express. 2025 Jul 21;15:108. doi: 10.1186/s13568-025-01916-3 (PMC12279650; doi:10.1186/s13568-025-01916-3)
Supplement: Supplementary file 2 — Supplementary Material 2. [file 13568_2025_1916_MOESM2_ESM.pdf]

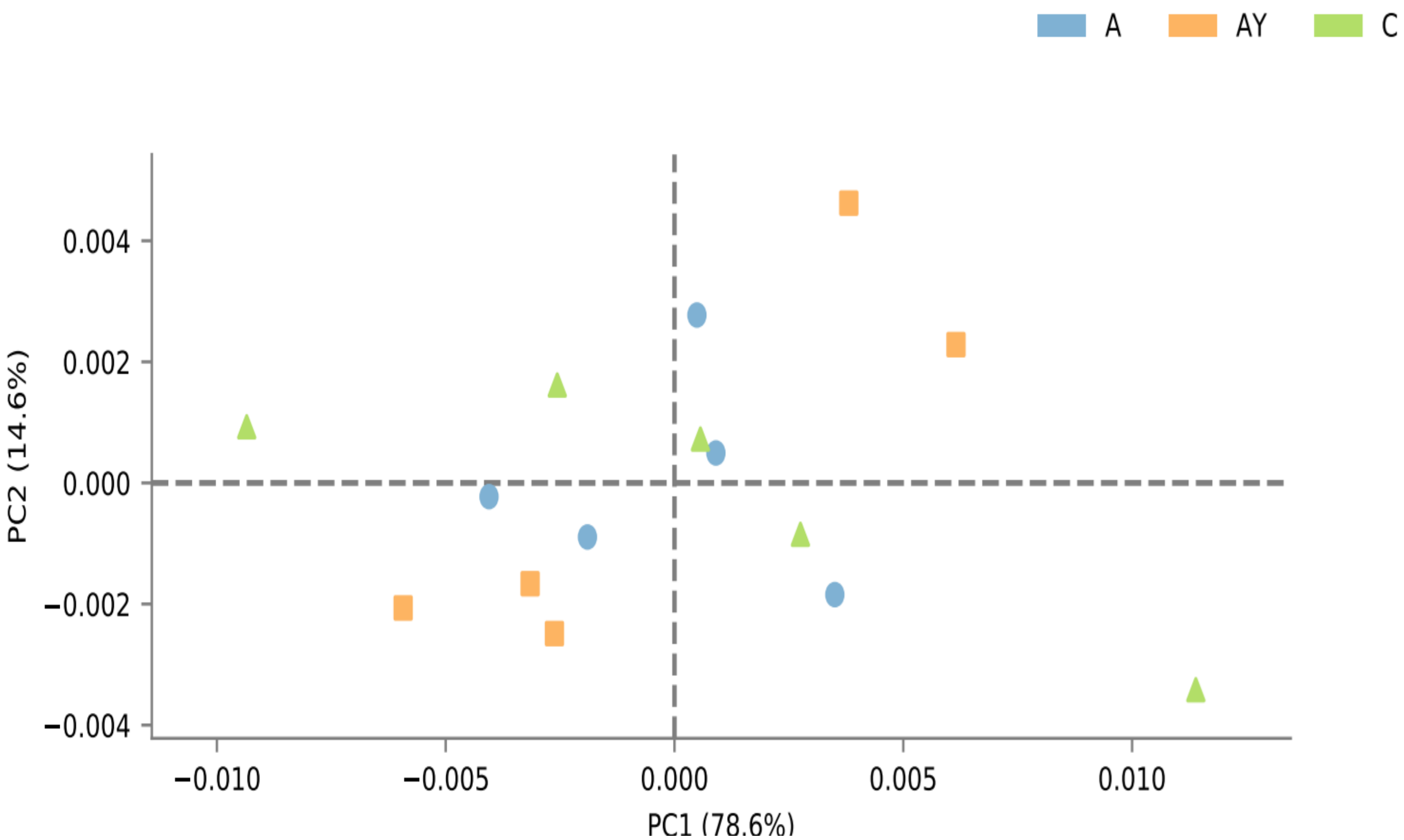

Supplementary figure S2: Principal components analysis of PICRUSt2 functional prediction of bacterial communities in the rumen of lactating goats supplemented with Spirulina and live yeast. Green triangles for control group (C), blue circles for group (A), and orange squares for group (AY).
