# Supplementary material for: Milk yield, rumen fermentation, and microbiota of Shami goats fed diets supplemented with spirulina and yeast
Source: AMB Express. 2025 Jul 21;15:108. doi: 10.1186/s13568-025-01916-3 (PMC12279650; doi:10.1186/s13568-025-01916-3)
Supplement: Supplementary file 4 — Supplementary Material 4. [file 13568_2025_1916_MOESM4_ESM.docx]

**Supplementary Table S1: Experimental treatments in in vitro and in vivo trials.**

| Diet | Supplementation type |
| --- | --- |
| **Experimental diets in *in vitro* trial** | |
| Control group (C) | 70% concentrate feed mixture (CFM) and 30% Alfalfa hay |
| Group Y | Control diet supplemented with 1% *Saccharomyces cerevisiae*) on the dry matter (DM) basis |
| Group A | Control diet supplemented with 1% *Spirulina platensis* on the dry matter (DM) basis |
| Group AY | Control diet supplemented with 1% a mixture of *Saccharomyces and* Spirulina (50% *Saccharomyces* and 50% *Spirulina*) on the dry matter (DM) basis |
| **Experimental diets in in vivo trial** | |
| Control group (C) | 70% concentrate feed mixture (CFM) and 30% Alfalfa hay |
| Group Y | Control diet supplemented with 1% *Saccharomyces cerevisiae*) on the dry matter (DM) basis |
| Group A | Control diet supplemented with 1% *Spirulina platensis* on the dry matter (DM) basis |
| Group AY | Control diet supplemented with 1% a mixture of *Saccharomyces and* Spirulina (50% *Saccharomyces* and 50% *Spirulina*) on the dry matter (DM) basis |
